# Alteration in mitochondrial Ca^2+^ uptake disrupts insulin signaling in hypertrophic cardiomyocytes

**DOI:** 10.1186/s12964-014-0068-4

**Published:** 2014-11-07

**Authors:** Tomás Gutiérrez, Valentina Parra, Rodrigo Troncoso, Christian Pennanen, Ariel Contreras-Ferrat, César Vasquez-Trincado, Pablo E Morales, Camila Lopez-Crisosto, Cristian Sotomayor-Flores, Mario Chiong, Beverly A Rothermel, Sergio Lavandero

**Affiliations:** Advanced Center for Chronic Disease (ACCDiS), Facultad de Ciencias Quimicas y Farmaceuticas & Facultad de Medicina, Universidad de Chile, Santiago, 838049 Chile; Department of Internal Medicine (Cardiology Division), University of Texas Southwestern Medical Center, Dallas, TX 75390-8573 USA; Instituto de Nutrición y Tecnología de los Alimentos (INTA), Universidad de Chile, Santiago, 7830490 Chile; Institute for Research in Dental Science, Faculty of Dentistry, Universidad de Chile, Santiago, 838049 Chile; Centro de Estudios Moleculares de la Célula, Facultad de Medicina, Universidad de Chile, Santiago, 838049 Chile

**Keywords:** Insulin, Calcium, Mitochondria, Cardiac hypertrophy, Inositol 1,4,5-triphosphate receptor, Akt, IGF-1, Catecholamines

## Abstract

**Background:**

Cardiac hypertrophy is characterized by alterations in both cardiac bioenergetics and insulin sensitivity. Insulin promotes glucose uptake by cardiomyocytes and its use as a substrate for glycolysis and mitochondrial oxidation in order to maintain the high cardiac energy demands. Insulin stimulates Ca^2+^ release from the endoplasmic reticulum, however, how this translates to changes in mitochondrial metabolism in either healthy or hypertrophic cardiomyocytes is not fully understood.

**Results:**

In the present study we investigated insulin-dependent mitochondrial Ca^2+^ signaling in normal and norepinephrine or insulin like growth factor–1-induced hypertrophic cardiomyocytes. Using mitochondrion-selective Ca^2+^-fluorescent probes we showed that insulin increases mitochondrial Ca^2+^ levels. This signal was inhibited by the pharmacological blockade of either the inositol 1,4,5-triphosphate receptor or the mitochondrial Ca^2+^ uniporter, as well as by siRNA-dependent mitochondrial Ca^2+^ uniporter knockdown. Norepinephrine-stimulated cardiomyocytes showed a significant decrease in endoplasmic reticulum-mitochondrial contacts compared to either control or insulin like growth factor–1-stimulated cells. This resulted in a reduction in mitochondrial Ca^2+^ uptake, Akt activation, glucose uptake and oxygen consumption in response to insulin. Blocking mitochondrial Ca^2+^ uptake was sufficient to mimic the effect of norepinephrine-induced cardiomyocyte hypertrophy on insulin signaling.

**Conclusions:**

Mitochondrial Ca^2+^ uptake is a key event in insulin signaling and metabolism in cardiomyocytes.

**Electronic supplementary material:**

The online version of this article (doi:10.1186/s12964-014-0068-4) contains supplementary material, which is available to authorized users.

## Background

Cardiac hypertrophy is a physiological process that occurs in response to an increase in heart workload. Initially, it can be compensatory, however, a chronic elevated workload, the neurohumoral input, or an underlying genetic mutation can lead to pathological hypertrophy, contractile dysfunction, ventricular dilatation, and finally, heart failure [1]. Moreover, during pathological hypertrophy, the heart undergoes significant changes in metabolism [2]. Cardiac metabolism is flexible, with fuel preferences switching from primarily glycolysis in the foetal heart, to β-oxidation of lipids in the adult heart [3]. During pathological hypertrophy, there is a re-activation of the so called “foetal gene program” and an increased preference for glucose oxidation [4,5].

The cellular signalling pathways involved in the hypertrophic response have been widely studied and Ca^2+^ is known to play a key role [1]. Diverse proteins activated by an elevation in cytosolic Ca^2+^ have been associated with the development of cardiac hypertrophy, such as the protein phosphatase calcineurin, Ca^2+^-calmodulin kinase II and protein kinase C [6-8]. Mitochondria are another important target of Ca^2+^ signalling. Mitochondrial Ca^2+^ uptake is essential for cell bioenergetics; thus an equilibrated rise in mitochondrial Ca^2+^ concentration stimulates Krebs cycle activity, increasing NADH levels and ATP synthesis [9,10]. Mitochondrial Ca^2+^ uptake is carried out primarily by the mitochondrial Ca^2+^ uniporter (MCU) [11]. This uniporter is a highly selective Ca^2+^ channel, but with low affinity [12]. Thereby, high cytosolic-Ca^2+^ concentrations in the proximity of mitochondria must occur to induce Ca^2+^ entry. To solve this problem, mitochondria are strategically localized to high Ca^2+^ concentration microdomains near Ca^2+^ release channels of the endoplasmic reticulum (ER) [13,14]. The close contacts formed between ER and mitochondria are regulated by a great variety of proteins located in the interface of the two organelles, including the inositol 1,4,5-triphosphate receptor (InsP_3_R), the ryanodine receptor (RyR), the sarco/endoplasmic reticulum Ca^2+^-ATPase (SERCA) and mitofusin-2 (Mfn2) [15,16].

Insulin is a key regulator of metabolism and plasma glucose levels [17]. In the heart, insulin promotes glucose uptake into cardiomyocytes and its use as an energy source by activating glycolysis and mitochondrial oxidative phosphorylation [18]. Recently, we showed that insulin increases cytoplasmic Ca^2+^ levels by a mechanism that involves the sequenced activation of phosphatidilinositol-3 kinase (PI3K) and phospholipase C (PLC) to increase the mass of InsP3. This axis induces Ca^2+^ release from the InsP_3_R, that can influence translocation of the facilitated glucose transporter GLUT4 to cell surface and glucose uptake [19]. Mitochondrial Ca^2+^ uptake can also affect cytoplasmic Ca^2+^ signals, by acting as a Ca^2+^ buffer after an increase in cytoplasmic levels. Moreover, it has been shown that, in addition to helping buffer large cytoplasmic Ca^2+^ fluctuations, the maintenance of optimal mitochondrial function is dependent on continual, constitutive Ca^2+^ transfer from ER to mitochondria to support oxidative phosphorylation [9]. During pathological cardiac hypertrophy, the heart develops insulin resistance, resulting in a slower rate of glucose entry in response to insulin and a decrease in the capacity for glucose oxidation [20,21], without changes in the expression of GLUT4 transporters [22].

The current study was designed to understand the mechanisms integrating mitochondrial Ca^2+^ uptake and insulin signaling in normal and hypertrophic cardiomyocytes. We show that insulin-induced stimulation of oxidative metabolism occurs through mitochondrial uptake of the Ca^2+^ released from ER. This mechanism is diminished in norepinephrine (NE)-treated cardiomyocytes undergoing pathological hypertrophy, but not in insulin like growth factor-1 (IGF-1)-treated hypertrophic cardiomyocytes. Moreover, pharmacological inhibition of mitochondrial Ca^2+^ uptake reduces insulin-dependent activation of the canonical Akt pathway. Thus, Ca^2+^ transfer to mitochondria emerges as an important new regulator of insulin signaling.

## Results

### Insulin induces an increase in mitochondrial Ca^2+^ uptake

Previous reports showed that insulin stimulation leads to an increase in cytoplasmic Ca^2+^ levels by releasing Ca^2+^ from the ER [19]. However, it is unknown whether the Ca^2+^ signal triggered by insulin has a mitochondrial component. To examine this, changes in mitochondrial Ca^2+^ levels were measured in cardiomyocytes using the mitochondrial-directed Ca^2+^ indicator Rhod-FF. This probe had a Pearson coefficient of 0.92 with the mitochondrial probe MitoTracker Green in colocalization analysis, showing a high degree of mitochondrial destination (Additional file 1: Figure S1A). Once loaded with Rhod-FF, changes in mitochondrial Ca^2+^ levels were measured via confocal microscopy. As a control, at the end of each measurement, carbonyl cyanide m-chlorophenyl hydrazone (CCCP) was added to dissipate mitochondrial membrane potential (ΔΨm) releasing mitochondria-localized Ca^2+^ to the cytoplasm (Additional file 1: Figure S1B). Because Rhod-FF at high concentrations can alter mitochondrial morphology [23] we also analyzed mitochondrial mean volume and number [24,25] in cells treated with MitoTracker Green and Rhod-FF (Additional file 1: Figure S1C-D). No significant differences were found in these parameters suggesting that under our conditions, treatment with Rod-FF did not impact mitochondrial morphology.

**Figure 1:**
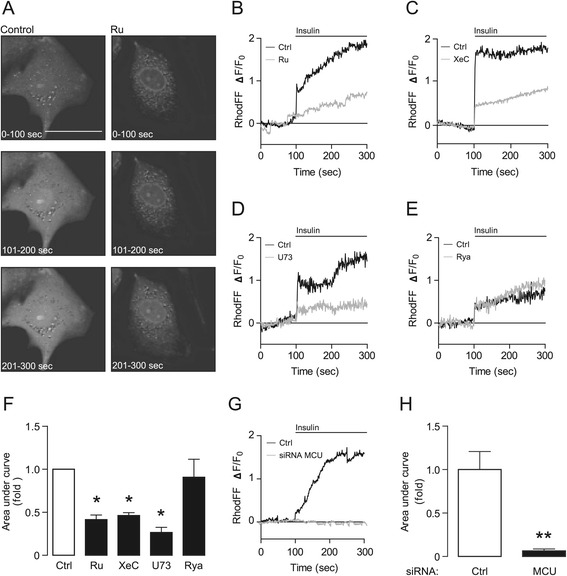
**Insulin increases mitochondrial Ca** ^**2+**^ **through an InsP**_**3**_ **R dependent pathway. A)** Confocal images of insulin-induced mitochondrial Ca^2+^ signal captured at the indicated times in cardiomyocytes loaded with Rhod-FF 5,4 μM. **B-E)** Representative measures of fluorescence-associated insulin-induced mitochondrial Ca^2+^ signals in cardiomyocytes pre-treated with (gray line) or without (Ctrl, black line) the respective inhibitors for 30 min. Insulin (100 nM) was added at 100 s. **B)** Ruthenium Red (Ru), 10 μM; **C)** xestospongin C (XeC), 100 μM; **D)** U73122 (U73), 10 μM and **E)** Ryanodine (Rya), 50 μM. **F)** Area under the curve of insulin-induced mitochondrial Ca^2+^ signals of control, Ru, XeC, U73 and Rya pre-treated cardiomyocytes after insulin stimulation between 100 and 300 s. The results are representative of 3 independent experiments (*N = 3*), where 10–20 cells were analysed. Data are expressed as mean ± SEM, *P < 0.05 vs. Ctrl. **G)** Measurement of insulin-induced mitochondrial Ca^2+^ signal in cardiomyocytes transfected with a siRNA control (black line) or a siRNA against MCU (gray line). Insulin (100 nM) was added at 100 s. **H)** Area under the curve of insulin-induced mitochondrial Ca^2+^ signals of control and siRNA-mCU treated cardiomyocytes. The results are representative of 3 independent experiments (N = 3), where 10–20 cells were analysed. Data are expressed as mean ± SEM, **P < 0.01 vs. siRNA Ctrl.

As shown in Figure 1A, mitochondrial Ca^2+^ levels increased in cardiomyocytes after insulin stimulation. Pharmacological inhibition was used to test the participation of various Ca^2+^ channels. It has been shown that MCU is one of the primary Ca^2+^ channels involved in mitochondrial Ca^2+^ uptake in cardiomyocytes [26]. To test whether MCU participates in insulin-induced mitochondrial Ca^2+^ uptake, cardiomyocytes were pre-treated with the MCU inhibitor Ruthenium Red (Ru) for 30 min prior insulin stimulation. As shown in Figure 1B, there was a significant reduction in mitochondrial Ca^2+^ uptake after insulin stimulation in Ru-treated cardiomyocytes. Next, participation of InsP_3_R was evaluated by pre-treating cardiomyocytes with an InsP_3_R–specific inhibitor xestospongin C (XeC). XeC also reduced the insulin-induced mitochondrial Ca^2+^ signal (Figure 1C). To further confirm the participation of InsP_3_R, PLC was blocked with U73122, thus inhibiting InsP_3_R Ca^2+^ release by preventing the synthesis of its ligand. Figure 1D shows that cardiomyocytes pre-treated with U73122 had a reduced mitochondrial Ca^2+^ signal after insulin stimulation. To evaluate whether RyR hannels also contribute to this mitochondrial Ca^2+^ signal, RyR channels were inhibited by preincubating cardiomyocytes with ryanodine. Figure 1E shows that the insulin-induced increase in mitochondrial Ca^2+^ was unaltered in cardiomyocytes pre-treated with ryanodine. In conclusion, Ru, XeC and U73122 pre-treatment caused a significant reduction in the area under the curve of the insulin-induced mitochondrial Ca^2+^ signal, whereas ryanodine did not (Figure 1F).

High concentrations of Ru can bind non-specifically to phospholipid membranes [27] potentially altering intracellular Ca^2+^ concentrations in our experiments. To control this, we used Fura2 to assess changes in cytoplasmic Ca^2+^ at baseline and after the addition of CCCP (50 μM) or thapsigargin (Thapsi, 500 μM) to deplete mitochondrial or ER Ca^2+^, respectively in cells treated with and without Ru (10 μM) for 3 h (Additional file 2: Figure S2A-C). No significant difference was found in basal cytoplasmic Ca^2+^ levels of cells treated with Ru in comparison with control cells, however, there was a slight, but not significant, decrease in the rate of Ca^2+^ increase (slope, [(F340/F380)/Time)] evoked with both stimuli, CCCP and Thapsi. Moreover, to further confirm the involvement of MCU in our model we used a complementary approach to alter MCU activity that was independent of any nonspecific effects of Ru. Cardiomyocytes were transiently transfected with a small interfering RNA (siRNA) against MCU, reaching a knockdown of 72%, 72 h post transfection (Additional file 2: Figure S2D-E). There was a significant reduction on insulin-induced mitochondrial Ca^2+^ uptake in siRNA-MCU depleted cells compared to cardiomyocytes transfected with a control siRNA (Figure 1G and H). Taken together, these results identify the insulin-induced increase in mitochondrial Ca^2+^ as a PLC/InsP_3_R/MCU dependent pathway.

### The Insulin-induced increase in mitochondrial Ca^2+^ is reduced in norepinephrine-treated hypertrophic cardiomyocytes

**Figure 2:**
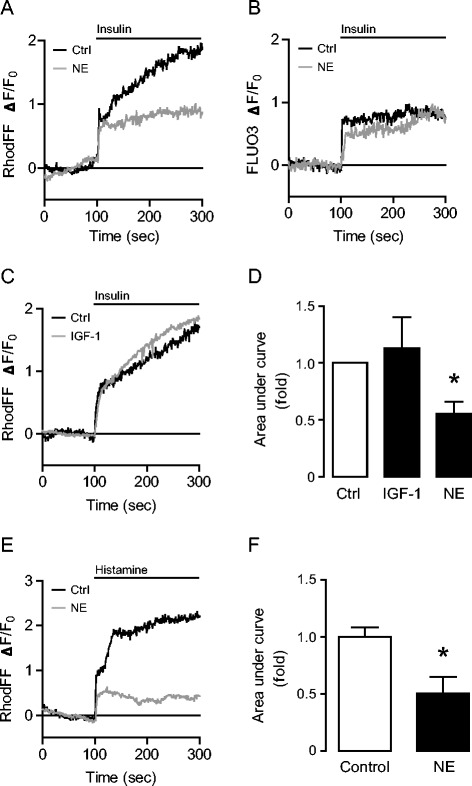
**Reduction in insulin-dependent mitochondrial Ca** ^**2+**^ **uptake in NE-induced hypertrophic cardiomyocytes.** Representative measures of fluorescence-associated insulin-induced Ca^2+^ signals in cardiomyocytes pre-treated with (gray line) or without (Ctrl, black line) the respective hypertrophic inducers for 24 h. Insulin (100 nM) was added at 100 s. **A)** Mitochondrial Ca^2+^ signals in cardiomyocytes treated with NE 10 μM for 24 h. **B)** Cytoplasmic Ca^2+^ signals in cardiomyocytes treated with NE 10 μM for 24 h. **C)** Mitochondrial Ca^2+^ signals in cardiomyocytes treated with IGF-1 100 nM for 24 h. **D)** Area under the curve of mitochondrial Ca^2+^ signals of control and IGF-1 and NE treated cardiomyocytes after insulin stimulation between 100 and 300 s. The results are representative of 3 independent experiments (N = 3), where 10–20 cells were analysed. Data are expressed as mean ± SEM, *P < 0.05 vs. Ctrl. **E)** Measurement of histamine-induced mitochondrial Ca^2+^ signal in cardiomyocytes treated with NE 10 μM for 24 h. Histamine (100 mM) was added at 100 s. **F)** Area under the curve of mitochondrial Ca^2+^ signals of control and NE treated cardiomyocytes after histamine stimulation between 100 and 300 s. The results are representative of 3 independent experiments (N = 3), where 10–20 cells were analysed. Data are expressed as mean ± SEM, *P < 0.05 vs. Ctrl.

During pathological hypertrophy, the heart develops significant changes in energy metabolism, shifting to an insulin resistant state [4]. To test whether the insulin-induced mitochondrial Ca^2+^ signal described here is altered in pathological hypertrophy, we evaluated this response in cardiomyocytes pre-treated with the adrenergic agonist NE (10 μM) for 24 h to induce hypertrophy (Additional file 3: Figure S3). Insulin-dependent mitochondrial Ca^2+^ uptake was reduced in the NE-treated cardiomyocytes compared to control cells (Figure 2A). Importantly, the insulin-induced increase in cytoplasmic Ca^2+^ levels was not reduced compared to controls in the NE-treated cardiomyocytes, as evaluated using Fluo3-AM (Figure 2B). Next, IGF-1-induced hypertrophy was used as an *in vitro* model of physiological hypertrophy [28]. Cardiomyocytes were treated with IGF-1 for 24 h prior to insulin stimulation. The mitochondrial Ca^2+^ signal induced by insulin was not reduced in IGF-1-treated cardiomyocytes compared to controls (Figure 2C and D), suggesting that the changes in mitochondrial Ca^2+^ uptake may be unique to the NE-induced pathological hypertrophy. To determine whether the reduction in mitochondrial Ca^2+^ uptake observed in NE-treated cardiomyocytes was due to a change upstream or downstream of Ca^2+^ release from ER, histamine was used to stimulate InsP_3_R-dependent Ca^2+^ release from the ER. Mitochondrial Ca^2+^ uptake after histamine stimulation was also reduced in NE-treated hypertrophic cardiomyocytes compared to control cells (Figure 2E and F). These results suggest that insulin-dependent mitochondrial Ca^2+^ uptake is lower in NE-treated cardiomyocytes (pathological hypertrophy) compared with control conditions. Importantly, this inhibitory effect appears not to be operative in IGF-1-treated cardiomyocytes (physiological hypertrophy) and thus is not simply a requisite change in response to cell growth. Moreover, histamine-dependent mitochondrial Ca^2+^ uptake was decreased in NE-treated cardiomyocytes, suggesting that changes in mitochondrial Ca^2+^ uptake in NE-treated cardiomyocytes occur downstream of Ca^2+^ release from ER and are not due to an upstream change in the insulin signaling. Interestingly, Ca^+2^ release from the ER after Thapsi treatment is augmented in NE-treated cardiomyocytes in comparison to control and IGF-1-treated cardiomyocytes, further indicating that the reduction in mitochondrial Ca^2+^ uptake is not due to a reduction in ER Ca^2+^ loading (Additional file 4: Figure S4).

### ER-mitochondria coupling is reduced in NE-treated hypertrophic cardiomyocytes

**Figure 3:**
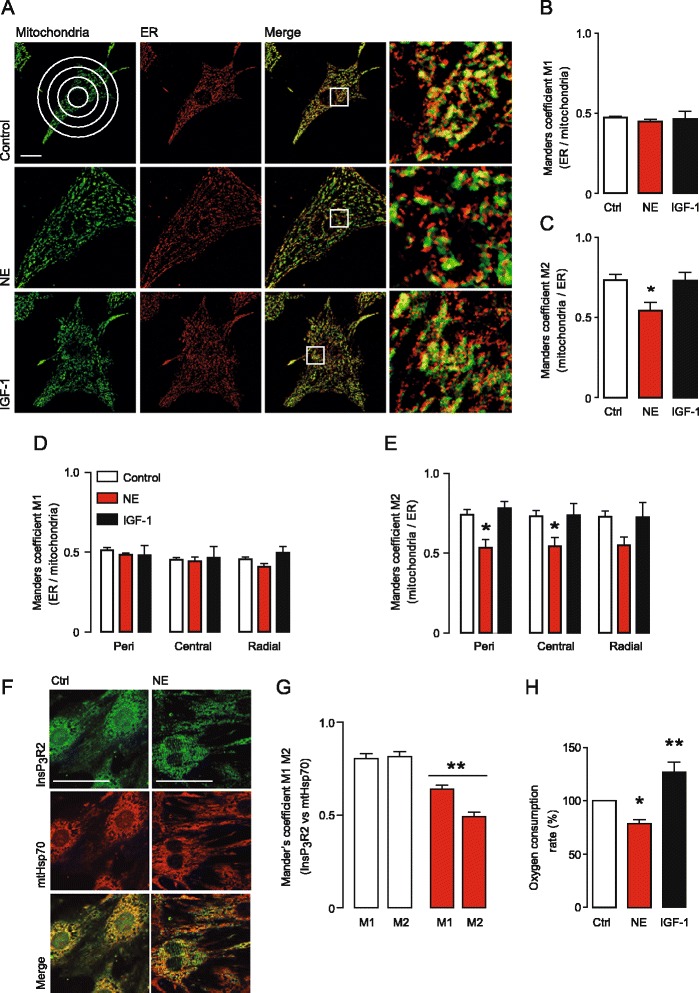
**ER-mitochondria coupling is reduced in NE-induced hypertrophic cardiomyocytes. A)** Representative confocal images of cardiomyocytes stained with MitoTracker green 200 nM for 30 min (Mitochondria, green) and ER-Tracker red 400 nM for 30 min (ER, red), treated with NE 10 μM or IGF-1 100 nM for 24 h. Scale bar: 10 μm. **B-C)** Quantification of the Manders’ coefficient M1 (fraction of ER in colocalization with mitochondria), or M2 (fraction of mitochondria in colocalization with ER). Data are expressed as mean ± SEM, N = 3, *P < 0.05 vs. Ctrl. **D-E)** Quantification of the M1 and M2 coefficients within the predefined subcellular regions [perinuclear (peri), central and radial]. Data are expressed as mean ± SEM, N = 3, *P < 0.05 vs. Control. **F)** Representative confocal images of cardiomyocytes stained for InsP_3_R2 (green) and mtHsp70 (red), treated with NE 10 μM for 24 h. Scale bar: 20 μm. **G)** Quantification of the Manders’ coefficient M1 (InsP_3_R2 overlapping mtHsp70) and M2 (mtHsp70 overlapping InsP_3_R2). Data are expressed as mean ± SEM, N = 3, **P < 0.01 vs. Ctrl. **H)** Oxygen consumption rate of cardiomyocytes treated with NE 10 μM or IGF-1 100 nM for 24 h. Data are expressed as mean ± SEM, N = 6, *P < 0.05 and **P < 0.01 vs. Control (ctrl).

The distance between ER and mitochondria is a key determinant of Ca^2+^ transfer between these organelles [29]. To evaluate whether this parameter was altered in the NE-treated cardiomyocytes, the colocalization of ER and mitochondria was measured using organelle specific fluorescent probes and confocal microscopy. Figure 3A shows representative images of control and NE or IGF-1 treated cardiomyocytes labeled with ER-Tracker red and MitoTracker green, to detect ER and mitochondria, respectively. The colocalization of these organelles was assessed in the whole cell by the quantification of Manders’ coefficient [30,31] which is a relative measure of the percentage of one structure in contact with other. The Manders’ coefficient M1 denotes the fraction of ER that colocalizes with mitochondria (Figure 3B), whereas the Manders' coefficient M2 denotes the fraction of mitochondria that colocalizes with ER (Figure 3C). M2 was significantly reduced in NE-treated cardiomyocytes compared with controls, indicating movement of mitochondria away from sites of contact with the ER. M1 did not change. Together with the Ca^2+^ uptake results, this data suggest there is a decrease in both physical and functional ER-mitochondrial coupling during pathologic hypertrophy. IGF-1-treated cardiomyocytes showed no change in either coefficient, consistent with maintenance of ER-mitochondria coupling during IGF-1-induced physiological hypertrophy. To further analyse the ER-mitochondria interaction, we measured the Manders’ coefficient using an algorithm for the ImageJ software previously developed by our group that is designed to scan the cell radially from the centre of the nucleus towards the plasma membrane in a full angle (0-360°), as illustrated in the upper left hand box of Figure 3A [31]. In this analysis, the control cells showed little variation in either Manders’ coefficient across the various regions of the cell, indicating that ER-mitochondria coupling is homogeneously distributed throughout the cell. Interestingly, this homogeneous distribution was maintained after either NE or IGF-1 treatment (Figure 3D and E). The reduction in M2 observed in NE-treated cardiomyocytes was observed in perinuclear, central, and radial regions, indicating that the reduction in ER-mitochondrial coupling occurs throughout the cardiomyocyte (Figure 3E). Because we previously identified InsP_3_R as the primary Ca^2+^ channel involved in Ca^2+^ transfer to the mitochondria, we evaluated the subcellular distribution of the most expressed InsP_3_R type 2 isoform (InsP_3_R2) and the mitochondrial marker mtHsp70. Figure 3F and G shows a reduction in the colocalization of InsP_3_R2 and the mitochondrial marker mtHsp70 in NE-treated cardiomyocytes compared to vehicle treated controls. In this case, both M1 and M2 were significantly reduced. We realize that there are important differences between the ER-mitochondrial interface in a functioning heart compared to cultured cells. Therefore a comprehensive validation of the differences between the two conditions is beyond the scope of our current manuscript, and although we believe that our findings are relevant to *in vivo* events, clearly further experimentation will be required to validate this.

Next, we tested if the reduction in ER-mitochondria coupling in NE-treated cardiomyocytes correlated with a change in mitochondrial metabolism. The rate of oxygen consumption was significantly reduced in NE-stimulated cardiomyocytes compared to controls. (78 ± 4, p <0.05) (Figure 3H). Oxygen consumption in IGF-1-stimulated cardiomyocytes was increased (127 ± 10, p <0.01) in comparison to control cells. All together, these data indicate that NE-induced pathologic hypertrophy causes a reduction in the physical association between ER and mitochondria and a reduction in mitochondrial metabolism, whereas IGF-1-induced physiologic hypertrophy does not. This reduction in physical contact between ER and mitochondria could be an underlying cause contributing to the reduction in insulin-stimulated mitochondrial Ca^2+^ uptake and mitochondrial metabolism seen under certain pathological hypertrophic states.

### Insulin signaling is blunted in NE-treated cardiomyocytes

**Figure 4:**
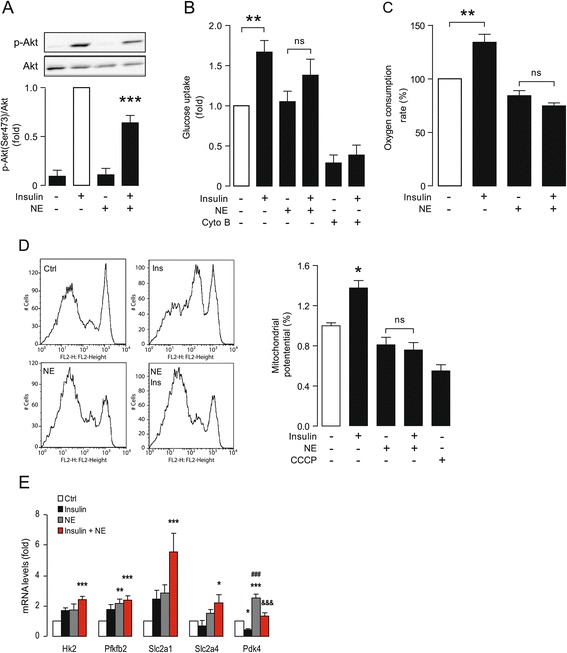
**Hypertrophic cardiomyocytes show a reduced insulin response. A)** Western blot of p-Akt (Ser^473^) and Akt (Upper) and densitometric analysis (lower) of control and hypertrophic NE-treated cardiomyocytes (NE 10 μM, 24 h) either stimulated with insulin 100 nM for 15 min or left unstimulated. Data were relativized against insulin (white bar) and expressed as mean ± SEM, N = 6, ***P < 0.001 vs. insulin. **B)** Glucose uptake of control and hypertrophic NE-treated cardiomyocytes (NE 10 μM for 24 h) either stimulated with insulin 100 nM for 15 min or left unstimulated. Cytochalasin B (Cyto B) was used as a negative control. Data are expressed as mean ± SEM, N = 7, **P < 0.01, ns: non significant. **C)** Oxygen consumption rates of control and hypertrophic cardiomyocytes (NE 10 μM for 24 h) either stimulated with insulin 100 nM for 3 h or left unstimulated. Data are expressed as mean ± SEM, N = 3, **P < 0.01 vs. control, ns: non significant. **D)** Mitochondrial membrane potential quantification in control cardiomyocytes or treated with NE 10 μM for 24 h and stimulated with insulin. CCCP (50 μM, 30 min) was used as a negative control. Data are expressed as mean ± SEM, N = 3, *P < 0.05 vs. control, ns: non significant. **E)** qPCR for Hk2, Pfkfb2, Slc2a1, Slc2a4 and Pdk4 mRNA levels of control (Ctrl) and hypertrophic cardiomyocytes (NE 10 μM for 24 h) either stimulated with insulin 100 nM for 3 h or left unstimulated. Data are expressed as mean ± SEM, N = 4, *P < 0.05, **P < 0.01 and ***P < 0.001 vs. Ctrl; ^*###*^ *P < 0.001* vs. Insulin and ^*&&&*^ *P < 0.001* vs. NE.

Pathological hypertrophy is associated with a decline in insulin sensitivity in both animal models and clinical populations with hypertrophic heart disease [20,21]. To determine whether canonical insulin signaling was altered in our *in vitro* cell culture model of NE-induced hypertrophy, various cellular processes regulated by insulin were measured. Akt is a serine/threonine kinase downstream of insulin receptor whose activity is regulated by phosphorylation [32]. Insulin treatment of control cardiomyocytes induced phosphorylation of Akt at Ser^473^ (Figure 4A). This response was reduced in hypertrophic cardiomyocytes pre-treated with NE, indicating a blunting of this early effector of insulin signaling. Consistent with this finding, insulin-stimulated glucose uptake was also reduced in the NE-treated cardiomyocytes (Figure 4B).

Previously, we and other groups demonstrated that insulin induces a rise in mitochondrial metabolism, that is fundamental to its ability to act as a metabolic regulator [25,33,34]. Therefore, we evaluated changes in mitochondrial respiration 3 h after insulin treatment by measuring oxygen consumption and mitochondrial membrane potential. Control cardiomyocytes showed a significant increase in oxygen consumption in response to insulin stimulation (134% ±8, p <0.01), whereas insulin did not significantly increase oxygen consumption in the NE-treated cardiomyocytes (Figure 4C). In parallel, mitochondrial membrane potential measured by flow cytometry, showed a very similar behaviour. Figure 4D shows that insulin increased mitochondrial membrane potential (138% ±10, p <0.05), whereas pre-treatment with NE completely abolished this response. Together, these results confirm that the metabolic response to insulin is reduced in NE-treated cardiomyocytes, consistent with reductions in insulin sensitivity reported for pathological hypertrophy *in vivo.* Because pyruvate dehydrogenase (PDH)-dependent glycolysis can be up-regulated in hypertrophy, we also analyzed the expression of five glycolytic genes (glycolytic markers): *hexokinase 2 (Hk2), 6-phosphofructo-2-kinase/fructose-2,6-biphosphatase 2 (Pfkfb2), Glut1 (Slc2a1), Glut4 (Slc2a4) and pyruvate dehydrogenase kinase, isozyme 4 (Pdk4)*. Figure 4E shows that expression of *Hk2*, *Pfkfb2*, *Slc2a1* and *Slca2a4* increase additively with insulin and NE treatments. However, in the case of *Pdk4*, a key enzyme down-regulated by insulin, NE had the opposite effect, increasing expression as well as preventing insulin-mediated downregulation. These data point to PDK4 regulation of PDH as a potentially interesting point of regulation between the glycolytic induction triggered by insulin and the chronic effects exerted by NE during hypertrophy.

### Blocking mitochondrial Ca^2+^ uptake mimics the hypertrophic metabolic phenotype

**Figure 5:**
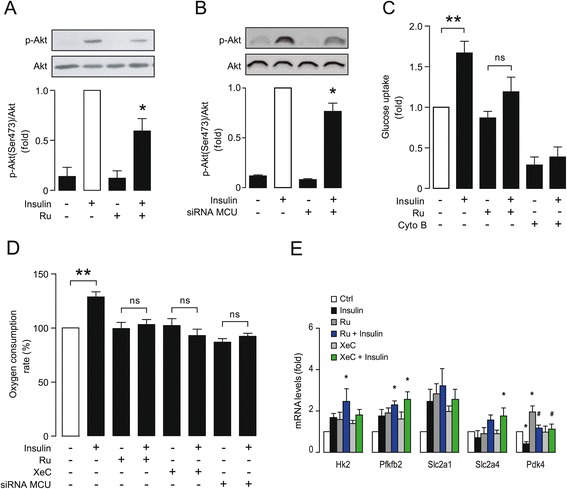
**The blockade of Ca** ^**2+**^ **entry to the mitochondria reduces insulin response. A)** Western blot of p-Akt (Ser^473^) and Akt (upper) and densitometric analysis (lower) of cardiomyocytes treated with ruthenium red (Ru 10 μM for 30 min) either stimulated with insulin (100 nM for 15 min) or left unstimulated. Data are expressed as mean ± SEM, N = 6, *P < 0.05 vs. insulin. **B)** Western blot of p-Akt (Ser^473^) and Akt (upper) and densitometric analysis (lower) of cardiomyocytes transfected with a siRNA Ctrl or against MCU either stimulated with insulin (100 nM for 15 min) or left unstimulated. Data are expressed as mean ± SEM, N = 3, *P < 0.05 vs. insulin. **C)** Glucose uptake of cardiomyocytes treated with ruthenium red (Ru 10 μM for 30 min) either stimulated with insulin 100 nM for 15 min or left unstimulated. Cytochalasin B (Cyto B) was used as a negative control. Data are expressed as mean ± SEM, N = 7, **P < 0.01 vs. control, ns: non significant. **D)** Oxygen consumption rates of cardiomyocytes treated with ruthenium red (Ru 10 μM, 30 min prior stimuli) or xestospongin C (XeC, 100 μM, 30 min prior stimuli) either stimulated with insulin (100 nM for 3 h) or left unstimulated. Data are expressed as mean ± SEM, N = 3, **P < 0.01 vs. control ns: non significant. **E)** qPCR for Hk2, Pfkfb2, Slc2a1, Slc2a4 and Pdk4 mRNA levels of cardiomyocytes treated with ruthenium red (Ru 10 μM for 30 min) or xestospongin C (XeC, 100 mM, 30 min prior stimuli) either stimulated with insulin (100 nM for 15 min) or left unstimulated. Data are expressed as mean ± SEM, N = 4, *P < 0.05 vs. Ctrl and ^*#*^ *P < 0.05* vs. Insulin.

To test for a causative relationship between changes in mitochondrial Ca^2+^ uptake and changes in insulin sensitivity, we pre-treated cardiomyocytes with Ru for 30 min to inhibit Ca^2+^ entry into mitochondria [35] and then evaluated the insulin-induced metabolic response of the cells. The response of Ru-treated cardiomyocytes was a phenotypic copy of NE-treated cardiomyocytes (Figure 4). Insulin-induced Ser^473^-Akt phosphorylation was reduced in cardiomyocytes with either Ru-treatment or siRNA-MCU knockdown (Figure 5A and B). Insulin-induced glucose uptake and stimulation of oxygen consumption were likewise reduced (Figure 5C-D). Furthermore, the insulin-induced increase in the oxygen consumption in cardiomyocytes was also blocked by pre-treatment with the InsP_3_R–specific antagonist XeC (which blocks Ca^2+^ release from the ER) as well as by depletion of MCU with siRNA (Figure 5D). Figure 5E shows the evaluation of the glycolytic genes assessed previously in Figure 4E. Consistent with the preceding data, insulin, Ru and XeC increased expression of the first four markers, whereas, Ru and XeC overcame the inhibitory effect of insulin on *Pdk4* expression.

Finally, to better understand the mechanism through which NE and mitochondrial Ca^2+^ uptake may be cross talk with insulin and Akt signalling, we assessed changes in phosphorylation of IRS-1 at Ser^307^. Phosphorylation of this site is inhibitory, uncoupling IRS-1 from the insulin receptor and decreasing activation of downstream targets including Akt [36]. A number of pathways can lead to Ser^307^ phosphorylation including insulin stimulation itself, thus providing feed back control over insulin stimulation. Consistent with decrease insulin-sensitivity in pathological hypertrophy [32] and the findings in Figure 4A, NE decreased insulin-induced phosphorylation of IRS-1 (Additional file 5: Figure S5). In this instance, the effect of Ru and XeC on insulin-stimulated Ser^307^ phosphorylation was less pronounced and not significant, suggesting that the impact of mitochondrial Ca^2+^ uptake on Akt signalling may not be the direct consequence of changes in insulin receptor sensitivity. This also highlights the complexity of the signalling mechanisms involved that merit further investigation.

**Figure 6:**
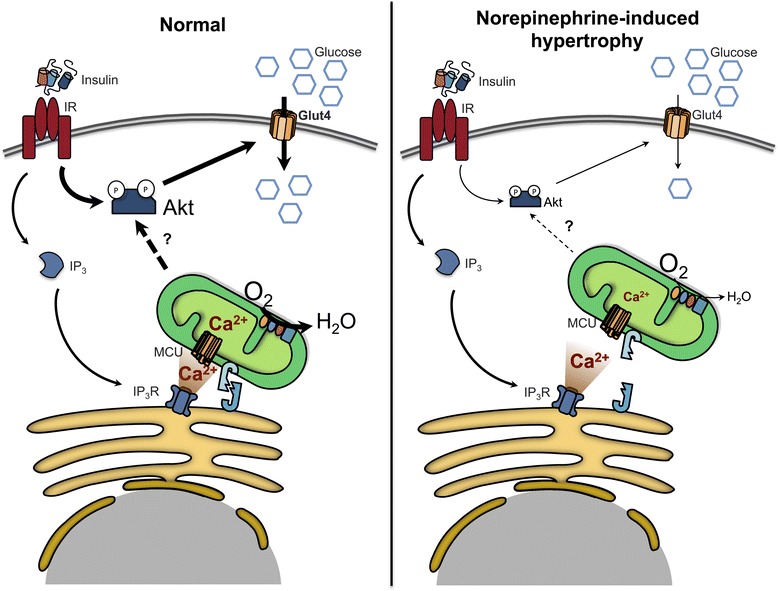
**Proposed model for insulin-induced mitochondrial Ca** ^**2+**^ **uptake in normal and hypertrophic cardiomyocytes.** During hypertrophy there is a decrease in ER-mitochondria physical coupling, disrupting Ca^2+^ transfers between both organelles, thus leading a diminished Akt phosphorylation and oxygen consumption in response to insulin.

Taken together, these data suggest that Ca^2+^ entry into mitochondria is an important aspect of the heart’s response to insulin, modulating both the activity of intermediates and the final metabolic effects at the level of cardiomyocytes. Furthermore, we show that this pathway of intracellular communication is altered in pathological, but not physiological hypertrophy and speculate that changes in this process could underlie the reduced insulin responses observed in other pathologies. The possible retrograde modulation of mitochondria to insulin signaling reveals an important new aspect of cellular crosstalk, placing mitochondria as an active participant in intracellular communication (Figure 6).

## Discussion

In recent decades there have been important advances in our understanding of the pathways mediating insulin signaling. The most widely studied is the canonical signaling cascade constituted by PI3K/Akt, that controls glucose uptake, metabolic activity, and translational responses to insulin [37]. Ca^2+^ has also been identified as an important second messenger acting downstream of the insulin receptor and shown to be essential in insulin-mediated glucose uptake in cardiac and skeletal muscle [38-40]. Our group showed that Ca^2+^ release from the ER through the InsP_3_R is an important component of the Ca^2+^-mediated insulin response in cardiomyocytes [19]. Mitochondria have a substantial capacity for Ca^2+^ storage that is facilitated by their physical and functional interaction with InsP_3_Rs in the ER membrane [41]. As such, poor or excessive mitochondrial Ca^2+^ retention represent a potential mechanism contributing to metabolic imbalance and insulin resistance.

The work presented here is the first to identify mitochondrial Ca^2+^ uptake as a key event in insulin-mediated signalling pathways in cardiomyocytes. We show that insulin-induced stimulation of oxidative metabolism via mitochondrial uptake of Ca^2+^ released from ER, whereas it is diminished in norepinephrine-treated cardiomyocytes undergoing pathological hypertrophy (Figure 6). Our results highlight the involvement of two intracellular Ca^2+^ channels: InsP_3_R, a principal Ca^2+^ ER channel, and MCU, which carries out mitochondrial Ca^2+^ uptake. The proximity of these two channels determines the efficiency of transfer of Ca^2+^ from ER to mitochondria.

### Mitochondrial insulin-dependent Ca^2+^ signals in hypertrophic cardiomyocytes

Mobilization of Ca^2+^ is known to facilitate insulin-stimulated glucose uptake in diverse cell types [38-40]. However, the precise mechanisms of action, the specific pools of Ca^2+^ involved, and how these phenomena may change under pathological conditions are largely unknown. Here, we evaluated both cytoplasmic and mitochondrial insulin-dependent Ca^2+^ signals in cardiomyocytes and assessed whether signaling is altered in hypertrophy. Deregulation of cytoplasmic Ca^2+^ levels and changes in the expression of various Ca^2+^ channels are hallmarks of cardiac hypertrophy [42-44]. However, with regards to cytoplasmic Ca^2+^ signals induced by insulin we found no alterations in the shape, timing or intensity in hypertrophic cardiomyocytes. This result, although surprising, does not preclude the possibility of alterations specific to particular cellular regions. In contrast, the mitochondrial Ca^2+^ response was blunted specifically in NE-treated cardiomyocytes, a model of pathological hypertrophy, but not in cardiomyocytes exposed to IGF-1, as a model of physiological hypertrophy (Figure 2). Mitochondrial Ca^2+^ uptake in response to histamine-mediated release of Ca^2+^ from the ER was likewise reduced in NE-treated cardiomyocytes compared to control or IGF-1 treated cardiomyocytes. This result demonstrates that the observed effects are not particular to insulin, but instead indicate a general decrease in the capacity for mitochondrial Ca^2+^ uptake in pathological hypertrophy. Our data are consistent with the work of Fauconnier *et al*. which described alterations in cytoplasmic and mitochondrial Ca^2+^ signaling in response to electrical stimulation in cardiomyocytes obtained from obese mice, another pathological condition where the heart develops insulin resistance [45]. In their study, using adult cardiomyocytes isolated from wild type and *ob/ob* mice, electrically evoked mitochondrial Ca^2+^ uptake was increased by insulin in cells from wild type, but not *ob/ob.* These findings suggest that, in addition to direct stimulation of mitochondrial Ca^2+^ uptake, insulin can increase the magnitude of uptake induced by other stimuli.

Our finding that blocking the entry of Ca^2+^ to mitochondria with Ru decreases insulin-dependent Akt phosphorylation suggests that this Ca^2+^ dynamic is relevant for proper insulin signaling to Akt. In this context, our group has previously demonstrated that inhibition of InsP_3_R with XeC and the Ca^2+^ chelating agent BAPTA-AM also reduces Akt phosphorylation [19]. Moreover, we showed that the induction of mitochondrial fragmentation reduces insulin-induced Akt phosphorylation by reducing mitochondrial Ca^2+^ uptake [35]. However, further investigation will be needed to fully understand the mechanisms involved in this signalling cross-talk. Taken together, these findings suggest that a reduction in the capacity of mitochondria to uptake Ca^2+^ may be a common feature of insulin resistance.

### ER-mitochondrial coupling in hypertrophic cardiomyocytes

Although the mechanism and regulatory components involved in mitochondrial Ca^2+^ uptake after ER release have been described, alterations in this process in pathological conditions are still unknown. The distance between ER and mitochondria is a key factor that determines the efficiency of Ca^2+^ transfer [46]. The ER and mitochondrial networks are highly dynamic and can change their shape and/or distribution in response to various stimuli. Bravo *et al*. showed that in early stages of ER stress, both mitochondria and ER migrate to the perinuclear region of the cell, increasing contacts between both organelles, enhancing Ca^2+^ transfer. This ultimately leads to an increase in mitochondrial metabolism [31]. We tested whether changes in ER-mitochondria coupling in pathological hypertrophy could underlie the observed decrease in insulin-stimulated mitochondrial Ca^2+^ uptake. Indeed, Figure 3 shows decreased colocalization and ER-mitochondrial coupling in pathological (NE) hypertrophied cardiomyocytes as compared to control or physiological (IGF-1) hypertrophied cardiomyocytes. The increased distance between the two-organelle populations would increase the potential for dispersion of Ca^2+^ released from the ER and prevent local Ca^2+^ concentrations at the ER-mitochondrial interface from reaching the critical concentration required for mitochondrial Ca^2+^ uptake via the MCU.

Our data show that reducing mitochondrial Ca^2+^ uptake with Ru is sufficient to confer an insulin-resistant phenotype with regards to Akt activation, glucose uptake, and oxygen consumption. Thus, mitochondrial Ca^2+^ uptake is not only a downstream target of insulin signaling, it also appears to participate in feed-forward signaling that helps to maintain insulin sensitivity.

## Conclusions

Mitochondrial Ca^2+^ uptake is a key event in insulin signalling and metabolism in cardiomyocytes. Our findings contribute to a better understanding of the causes that trigger insulin resistance in pathological hypertrophy. This work raises important new questions that place mitochondrial Ca^2+^ handling at a nodal point of metabolic control and demonstrate that preservation of this function is essential for proper cardiac function.

## Methods

### Culture and treatment of cardiomyocytes

Neonatal ventricular cardiomyocytes were prepared from hearts of 1-3-day-old Sprague Dawley rats as described previously [47]. They were euthanized by decapitation. Ventricles were trisected, pooled, and cardiomyocytes dissociated in a solution of collagenase and pancreatin. After enzymatic dissociation, cells were selectively enriched for cardiomyocytes by being pre-plated in DMEM/M199 (4:1) containing 10% (v/v) foetal bovine serum, 5% (v/v) foetal calf serum, penicillin, and streptomycin (100 units/ml). Cardiomyocytes were plated at a density of 2.5x10^5^ or 1.5x10^6^ for microscopy or Western blot experiments respectively. Serum was withdrawn for 24 h before the cells were treated with insulin (100 nM) and mitochondrial and cytoplasmic Ca^2+^ were assessed. In some experiments, cardiomyocyte hypertrophy was induced with NE (10 μM) or IGF-1 (100 nM) for 24 h and then stimulated with insulin. To inhibit the mitochondrial Ca^2+^ uniporter, Ru and siRNA to MCU were used. The InsP_3_R, ryanodine receptor and PLC were inhibited with XeC, ryanodine and U73122, respectively. Studies were approved by the Institutional Bioethical Committee, Faculty of Chemical and Pharmaceutical Sciences, University of Chile, in accordance with the National Institutes of Health *Guide for the Care and Use of Laboratory Animals* published by the US (NIH Publication, 8th Edition, 2011).

### Cardiomyocyte transfection

Small interfering RNAs (siRNAs) for MCU I and II and negative control (MISSION; Sigma-Aldrich Co.) were used according to manufacturer’s instructions. The siRNAs used for knockdown experiments were as follows: negative control, catalogue number SIC001; MCU I, sense (5′-CAGAGACCCUGAACGAUGU-3′), antisense (5′-ACAUCGUUCAGGGUCUCUG-3′); and MCU II, sense (5′-GGCUUACCUGGUGGGAAUA-3′), antisense (5′-UAUUCCCACCAGGUAAGCC-3’). Both siRNAs were tested, obtaining the best results with the siRNA that we called II, which was the one used for all the subsequent experiments (Additional file 2: Figure S2).

### Intracellular and mitochondrial Ca^2+^ determinations

Cytosolic Ca^2+^ levels were determined in cardiomyocytes preloaded with Fluo3-AM (5.4 μM, 30 min) or Fura2 (5 μM, 30 min), as described previously [19,48]. To determine mitochondrial Ca^2+^ levels, images were obtained from cultured cardiomyocytes preloaded with Rhod-FF (5.4 μM, 30 min) [49,50]. At the end of each measurement, CCCP 10 μM was used as control. Both determinations were performed in an inverted confocal microscope (Carl Zeiss LSM-5, Pascal 5 Axiovert 200 microscope).

### ER and mitochondrial network imaging

For ER and mitochondrial imaging, cells were treated during 30 min with 200 nM ER-Tracker red and with 400 nM MitoTracker Green, respectively. Confocal image stacks were captured with an inverted confocal microscope (Carl Zeiss LSM-5, Pascal 5 Axiovert 200 microscope).

### Image processing

For mitochondrial network and ER colocalization, one focal plane was analyzed. The images obtained were deconvolved using the ImageJ software. Colocalization between organelles was quantified using the Manders’ algorithm, as previously described [24,30,31,51]. For each independent experiment, 10–15 cells were registered.

### Mitochondrial dynamics analysis

Cells were incubated for 30 min with MitoTracker Green FM (400 nM) with or without Rhod-FF (5.4 μM) and maintained in Krebs solution. Confocal image stacks were captured with a Leica TCS SP5 confocal microscope and a Plan-APOCHROMAT 63x/1.4 Oil DIC objective as previously described [24,25]. Images were deconvolved with ImageJ software (National Institutes of Health), and volume reconstitution of Z-stacks of thresholded images was performed. The number and individual volume of each object (mitochondria) were quantified with the ImageJ 3D Object Counter plug-in. Each experiment was done at least three times, and 16–25 cells per condition were quantified.

### Western blot

The primary antibodies used were anti-β-MHC (1:1000, Sigma-Aldrich), anti-phospho-Akt (Ser^473^) (1:1000, Cell Signaling), anti-Akt (1:1000, Cell Signaling), anti IRS-1 (1:1000, Cell Signaling), anti-phospho-IRS-1 (Ser^307^) (1:500, Cell Signaling) and anti-MCU (1:2000, Abcam). Then the blots were incubated with a horseradish peroxidase-coupled secondary antibody (1:5000, Pierce). ImageJ software was used for image densitometry.

### Oxygen consumption

Cardiomyocytes were plated and treated according each experiment. Cells were then trypsinized, and the suspension was placed in a chamber at 25°C, coupled to a Clark electrode 5331 (Yellow Springs Instruments) where the oxygen consumption was measured polarographically [31,50].

### Glucose uptake

Cardiomyocytes were rinsed twice with HEPES-buffered saline. Inhibitors were added for 30 min and insulin during the last 10 min. Glucose uptake was measured using 10 mM [^3^H]2-deoxyglucose [19].

### Flow cytometry analysis of mitochondrial membrane potential

ΔΨm was measured after loading cardiomyocytes with TMRM (200 nM) for 30 min. Afterward, cells underwent trypsinization, and fluorescence was assessed by flow cytometry (excitation/emission 543/560) with a FACScan system (Becton Dickinson). CCCP (50 mM) and oligomycin (10 mM) for 30 min were used as positive and negative controls for the ΔΨm measurements.

### qPCR

Real-time PCR was performed with SYBR green (Applied Biosystems) as previously described [25]. Data for each transcript was normalized to 18S rRNA as internal control with the 2-ΔΔCt method. Primers used were as follows: MCU rat forward 5′-GCGCCAGGAATATGTTTATC-3′; MCU rat reverse 5′-TTGCATCCTTGAGTTGATTG-3′; Hk2 rat forward 5′-CGAATCAAAGAGAACAAGGG-3′; Hk2 rat reverse 5′-CAAAATGGGGATGTTTCTTG-3′; Pfkfb2 rat forward 5′-CTGGAGGTAAAAGTGTCAAG-3′; Pfkfb2 rat reverse 5′-ACGAGAGGTCCTTATCATAG-3′; Slc2a1 rat forward 5′-CAATATGTGGAGCAACTGTG-3′; Slc2a1 rat reverse 5′-AGTAGGTGAAGATGAAGAAGAG-3′; Slc2a4 rat forward 5′-AAGTGATTGAACAGAGCTAC-3′; Slc2a4 rat reverse 5′-CTTTTCCTTCCCAACCATTG-3′; Pdk4 rat forward 5′-AATCAAGATTTCTGACCGAG-3′; Pdk4 rat reverse 5′-CTGACATGGAATAGAGATTCAG-3′.

### Statistical analysis

Data are presented either as means ± SEM of a number (n) of independent experiments or as examples of representative experiments performed on at least three separate occasions. Data were analysed by analysis of variance and comparisons between groups were performed using a protected Tukey *t* test. A value of *P < 0.05* was chosen as the limit of statistical significance.

## Additional files

Additional file 1: Figure S1. Measurement of Rhod-FF fluorescence for Ca^2+^ signalling in cardiomyocytes. **A)** Representative confocal images stained with MitoTracker green (green, top left) and Rhod-FF (red, top right). The merge between MitoTracker green and Rhod-FF is shown in the down left corner and the intensity correlation analysis and the Pearson coefficient is shown in the down right corner. Scale bar: 10 μm. **B)** Representative measurement of Rhod-FF fluorescence representing the insulin-induced mitochondrial Ca^2+^ signal. Insulin (100 nM) was added at 100 s, and CCCP (50 μM) was added at 350 s. **C)** Representative confocal images of cells loaded with MitoTracker Green (400 nM for 30 min) alone, or MitoTracker Green (400 nM for 30 min) and Rhod-FF (5.4 μM for 30 min). Scale bar represents 10 μm. **D)** Number of mitochondria per cell and individual mitochondrial volume were determined for the cells in C). Data are expressed as mean ± SEM (n = 3). Additional file 2: Figure S2. Ruthenium Red (Ru) and MCU siRNA knockdown controls. **A)** Representative measures of Fura2 fluorescence-associated CCCP (50 μM) or thapsigargin (Thapsi, 500 μM) -induced cytosolic Ca^2+^ signals in cardiomyocytes pre-treated with (red line) or without (Ctrl, black line) Ruthenium Red (Ru, 10 μM) for 3 h. **B)** Basal Fura2 Fluorescence previous to CCCP or Thapsi stimuli. **C)** Initial Ca^2+^-related fluorescence rate (slope) of control and Ru (10 μM) pre-treated cardiomyocytes after CCCP (50 μM) or Thapsi (500 μM) stimulation at 150 s. The results are representative of 4 independent experiments (N =  4), where 10–20 cells were analysed. Data are expressed as mean ± SEM. **D)** qPCR for MCU mRNA levels of cardiomyocytes transfected with control, MCU (I) or MCU (II) siRNA. **E)** Western blot of MCU and β-tubulin (upper) and densitometric analysis (lower) of cardiomyocytes transfected with control, MCU (I) or MCU (II) siRNA. Data are expressed as mean ± SEM, N = 3, **P < 0.05*, ***P < 0.01* and ****P < 0.001* vs. siRNA Control (Ctrl). Additional file 3: Figure S3 Cardiomyocyte hypertrophy induced by NE. **A)** Representative images of cultured neonatal rat cardiomyocytes treated with or without (Control) NE 10 μM for the indicated times. Cells were fixed and stained with phalloidin-rhodamine (orange) for sarcomere detection and with Höescht (blue) for nuclei detection. Lower panels show the fluorescence intensity profiles of the lines depicted in the respective images. Scale bar: 20 μm. **B)** Cell area, **C)** perimeter and **D)** percentage of sarcomeric cardiomyocytes were calculated measuring at least 100 cells per condition. **E)** Western blot of β-MHC and β-actin (upper) and densitometric analysis (lower) of cardiomyocytes treated with or without NE 10 μM for the indicated times. Data are expressed as mean ± SEM, N = 3, **P < 0.05*, ***P < 0.01* and ****P < 0.001* vs Control. Additional file 4: Figure S4. ER Ca^2+^ content in hypertrophic cardiomyocytes. **A)** Cytoplasmic Ca^+2^ signals induced by thapsigargin 500 μM in control and NE 10 μM or IGF-1 100 nM treated cardiomyocytes. Caffeine 5 mM was added as a negative control. **B)** Area under the curve of cytoplasmic Ca^2+^ signals of control and NE and IGF-1 treated cardiomyocytes after thapsigargin 500 μM stimulation between 100 and 300 s. Data are expressed as mean ± SEM, N = 3, ***P < 0.01* vs Control. Additional file 5: Figure S5. IRS-1 phosphorylation in hypertrophic cardiomyocytes. **A)** Western blot of p-IRS-1 (Ser^307^) and IRS-1 (left) and densitometric analysis (right) of control and hypertrophic NE treated cardiomyocytes (NE 10 μM, 24 h) either stimulated with insulin 100 nM for 15 min or left unstimulated. Data were relativized against insulin (white bar) and expressed as mean ± SEM, N = 4, ****P < 0.001* vs. insulin. **B)** Western blot of p-IRS-1 (Ser^307^) and IRS-1 of control, Ruthenium Red (Ru, 10 μM, 30 min prior stimuli) and xestospongin C (XeC, 100 μM, 30 min prior stimuli) treated cardiomyocytes either stimulated with insulin 100 nM for 15 min or left unstimulated. Data were relativized against insulin (white bar) and expressed as mean ± SEM, N = 3, **P < 0.001* vs. Insulin.
