# Supplementary figures and images for: Alteration in mitochondrial Ca2+ uptake disrupts insulin signaling in hypertrophic cardiomyocytes
Source: Cell Commun Signal. 2014 Nov 7;12:68. doi: 10.1186/s12964-014-0068-4 (PMC4234850; doi:10.1186/s12964-014-0068-4)

# Additional file 1: Figure S1.

A

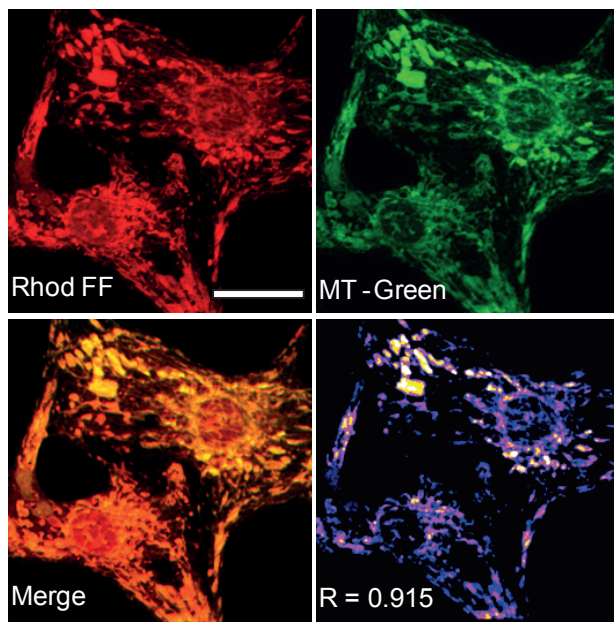

B

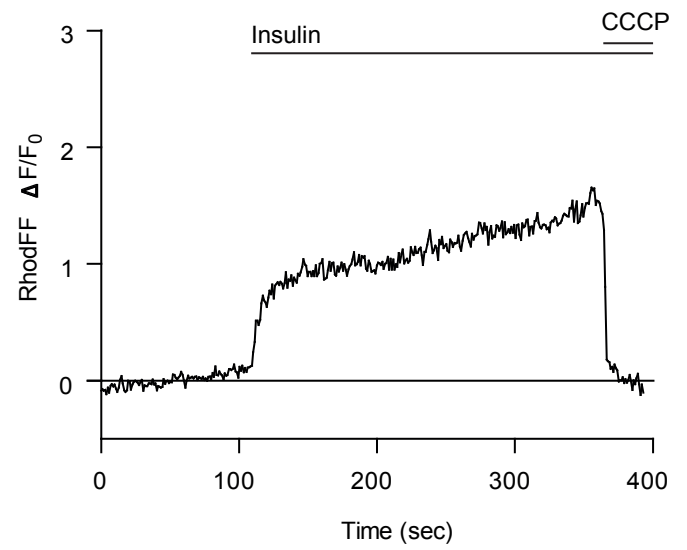

C

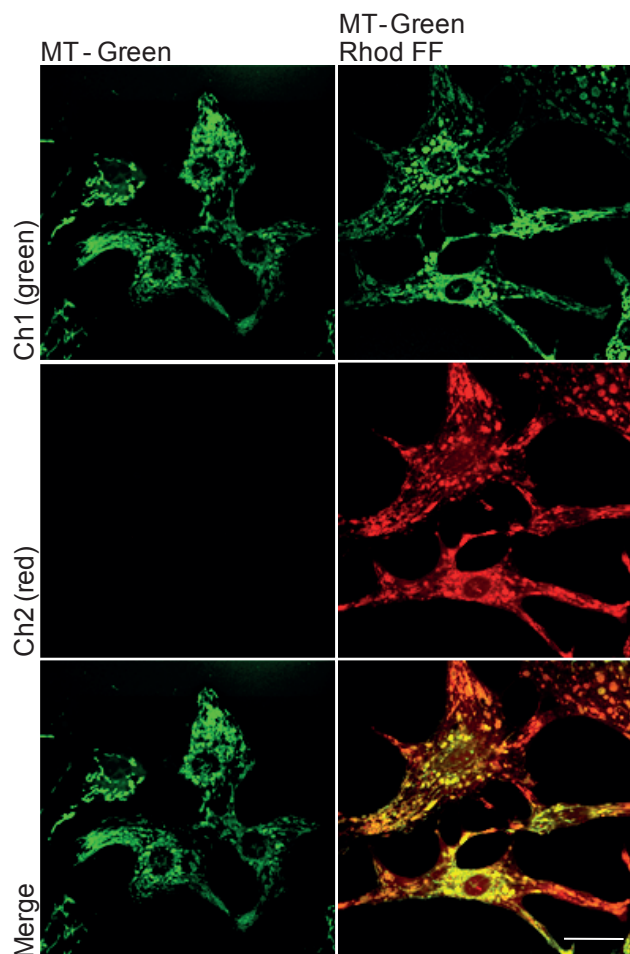

D

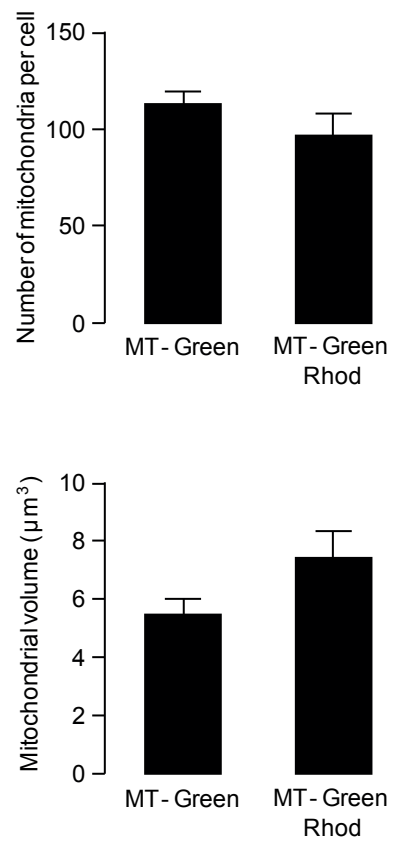

Supplement: Additional file 1: Figure S1. — Measurement of Rhod-FF fluorescence for Ca2+ signalling in cardiomyocytes. A) Representative confocal images stained with MitoTracker green (green, top left) and Rhod-FF (red, top right). The merge between MitoTracker green and Rhod-FF is shown in the down left corner and the intensity correlation analysis and the Pearson coefficient is shown in the down right corner. Scale bar: 10 μm. B) Representative measurement of Rhod-FF fluorescence representing the insulin-induced mitochondrial Ca2+ signal. Insulin (100 nM) was added at 100 s, and CCCP (50 μM) was added at 350 s. C) Representative confocal images of cells loaded with MitoTracker Green (400 nM for 30 min) alone, or MitoTracker Green (400 nM for 30 min) and Rhod-FF (5.4 μM for 30 min). Scale bar represents 10 μm. D) Number of mitochondria per cell and individual mitochondrial volume were determined for the cells in C). Data are expressed as mean ± SEM (n = 3). [file 12964_2014_68_MOESM1_ESM.pdf]

## Additional file 2: Figure S2.

A

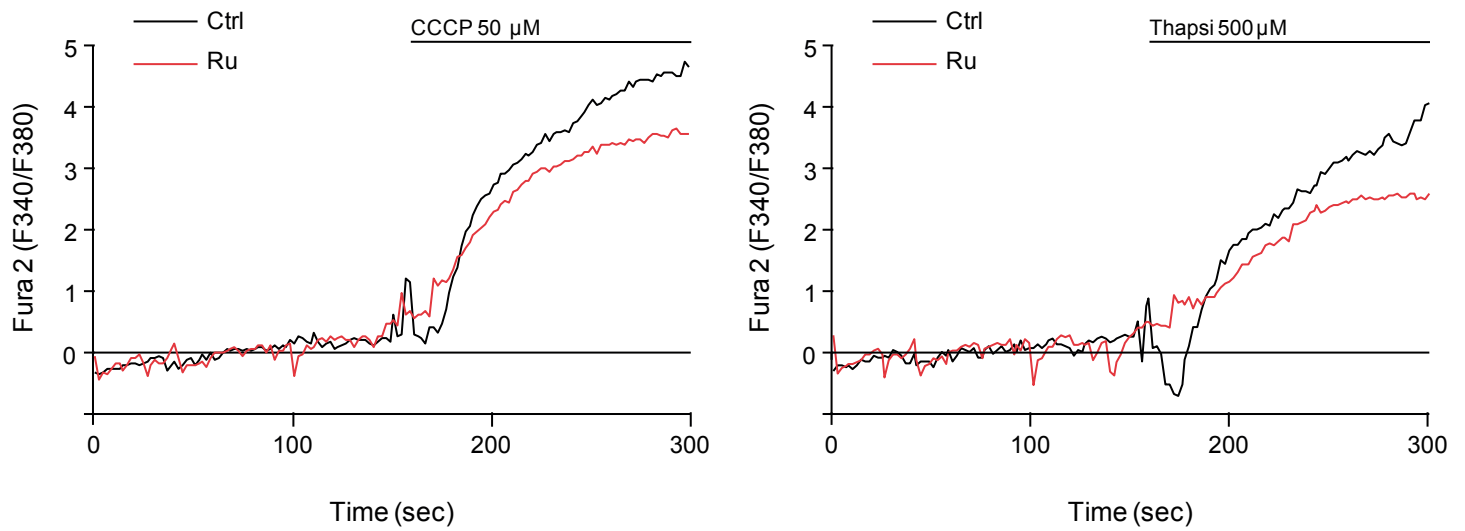

B

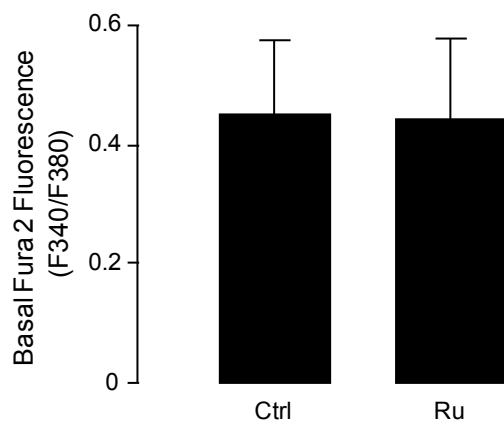

C

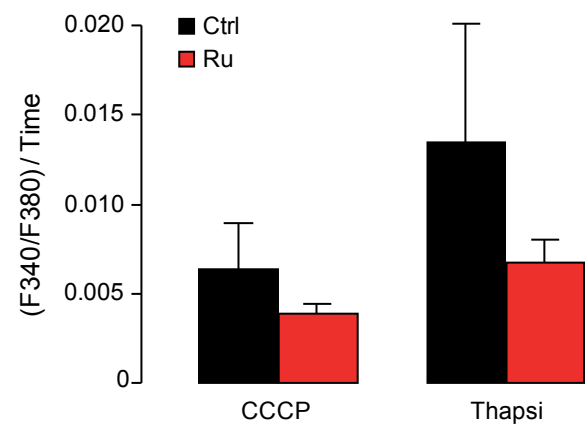

D

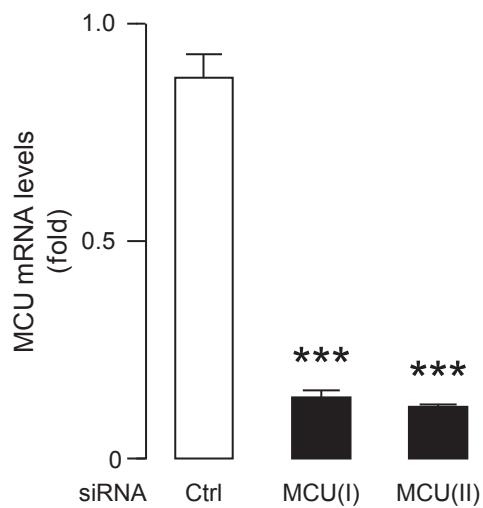

E

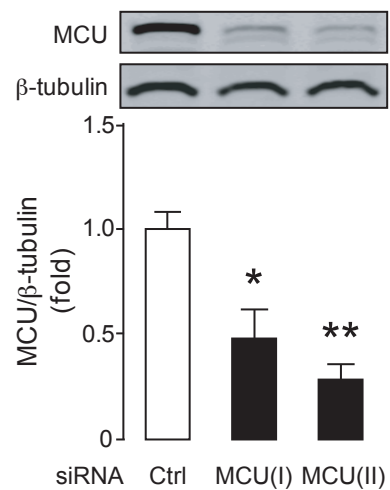

Supplement: Additional file 2: Figure S2. — Ruthenium Red (Ru) and MCU siRNA knockdown controls. A) Representative measures of Fura2 fluorescence-associated CCCP (50 μM) or thapsigargin (Thapsi, 500 μM) -induced cytosolic Ca2+ signals in cardiomyocytes pre-treated with (red line) or without (Ctrl, black line) Ruthenium Red (Ru, 10 μM) for 3 h. B) Basal Fura2 Fluorescence previous to CCCP or Thapsi stimuli. C) Initial Ca2+-related fluorescence rate (slope) of control and Ru (10 μM) pre-treated cardiomyocytes after CCCP (50 μM) or Thapsi (500 μM) stimulation at 150 s. The results are representative of 4 independent experiments (N = 4), where 10–20 cells were analysed. Data are expressed as mean ± SEM. D) qPCR for MCU mRNA levels of cardiomyocytes transfected with control, MCU (I) or MCU (II) siRNA. E) Western blot of MCU and β-tubulin (upper) and densitometric analysis (lower) of cardiomyocytes transfected with control, MCU (I) or MCU (II) siRNA. Data are expressed as mean ± SEM, N = 3, *P < 0.05, **P < 0.01 and ***P < 0.001 vs. siRNA Control (Ctrl). [file 12964_2014_68_MOESM2_ESM.pdf]

Additional file 3: Figure S3.

A

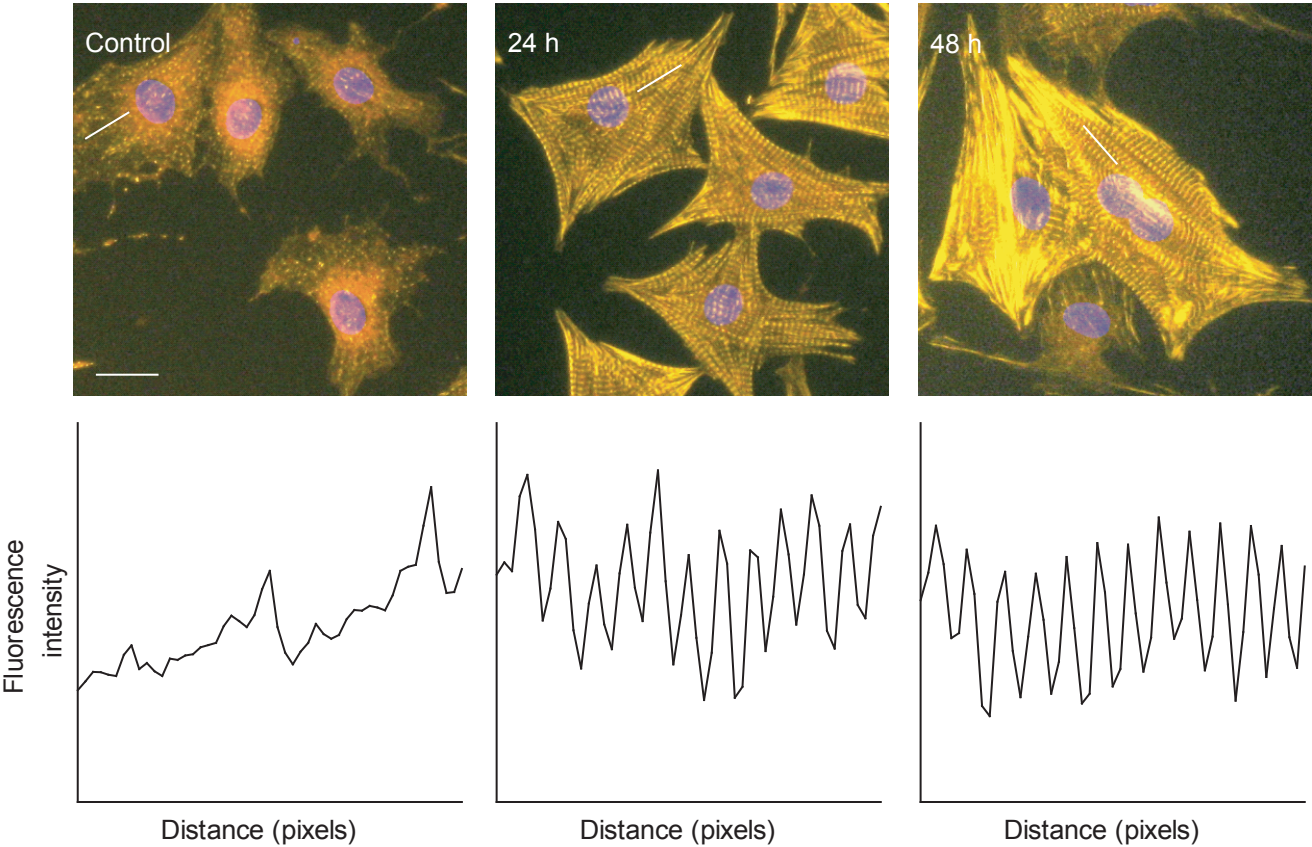

B

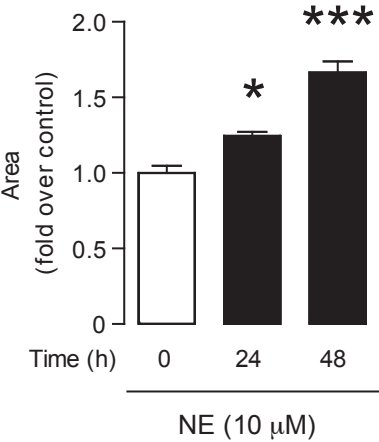

C

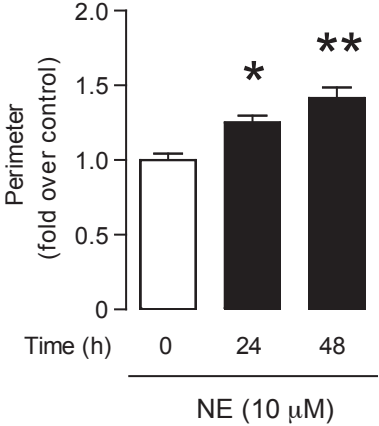

D

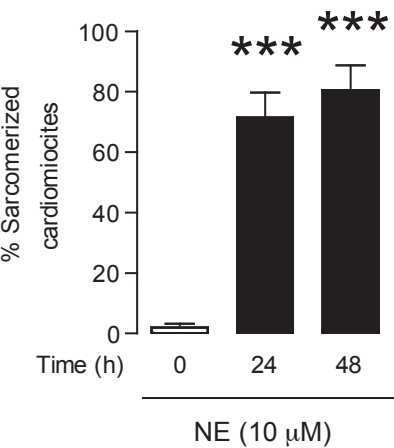

E

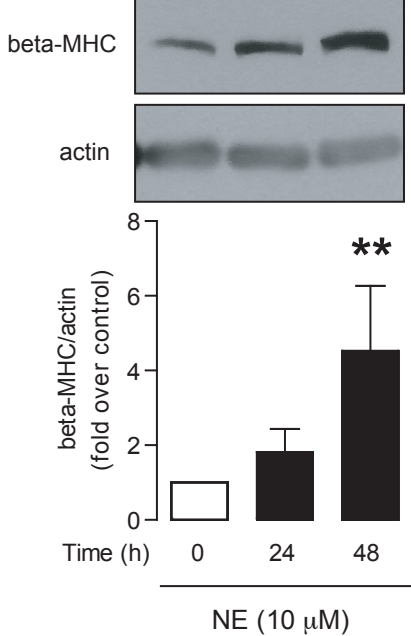

Supplement: Additional file 3: Figure S3 — Cardiomyocyte hypertrophy induced by NE. A) Representative images of cultured neonatal rat cardiomyocytes treated with or without (Control) NE 10 μM for the indicated times. Cells were fixed and stained with phalloidin-rhodamine (orange) for sarcomere detection and with Höescht (blue) for nuclei detection. Lower panels show the fluorescence intensity profiles of the lines depicted in the respective images. Scale bar: 20 μm. B) Cell area, C) perimeter and D) percentage of sarcomeric cardiomyocytes were calculated measuring at least 100 cells per condition. E) Western blot of β-MHC and β-actin (upper) and densitometric analysis (lower) of cardiomyocytes treated with or without NE 10 μM for the indicated times. Data are expressed as mean ± SEM, N = 3, *P < 0.05, **P < 0.01 and ***P < 0.001 vs Control. [file 12964_2014_68_MOESM3_ESM.pdf]

Additional file 4: Figure S4.

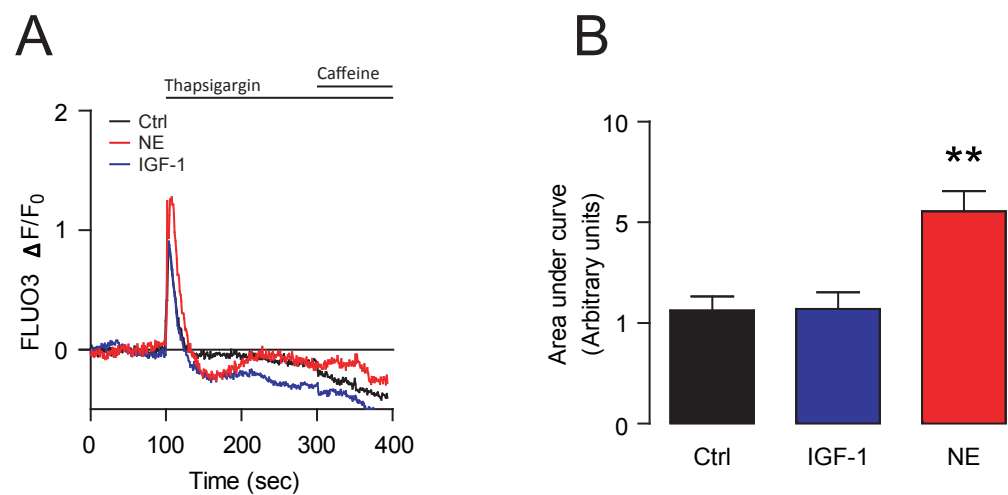

Supplement: Additional file 4: Figure S4. — ER Ca2+ content in hypertrophic cardiomyocytes. A) Cytoplasmic Ca+2 signals induced by thapsigargin 500 μM in control and NE 10 μM or IGF-1 100 nM treated cardiomyocytes. Caffeine 5 mM was added as a negative control. B) Area under the curve of cytoplasmic Ca2+ signals of control and NE and IGF-1 treated cardiomyocytes after thapsigargin 500 μM stimulation between 100 and 300 s. Data are expressed as mean ± SEM, N = 3, **P < 0.01 vs Control. [file 12964_2014_68_MOESM4_ESM.pdf]

Additional file 5: Figure S5.

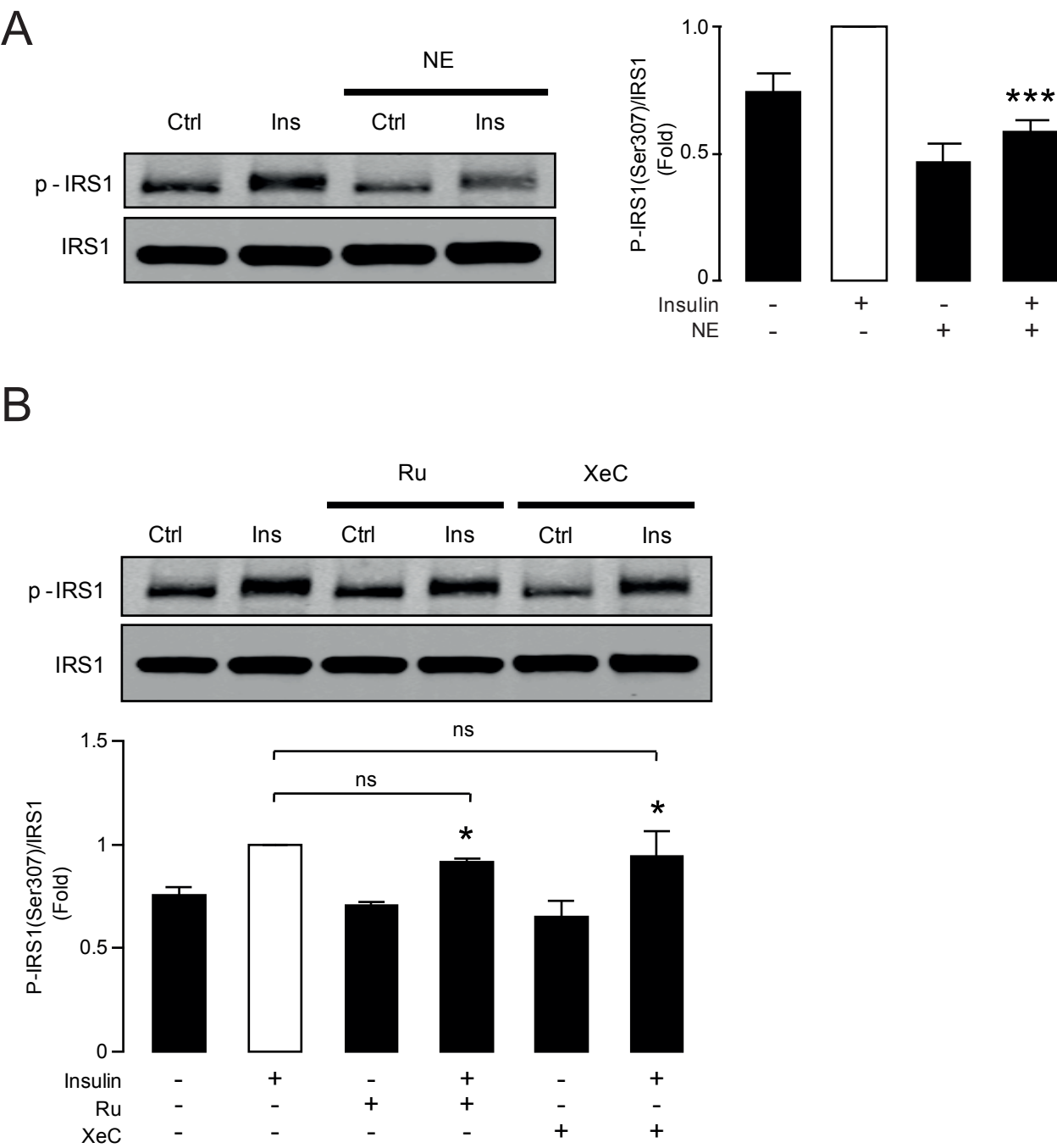

Supplement: Additional file 5: Figure S5. — IRS-1 phosphorylation in hypertrophic cardiomyocytes. A) Western blot of p-IRS-1 (Ser307) and IRS-1 (left) and densitometric analysis (right) of control and hypertrophic NE treated cardiomyocytes (NE 10 μM, 24 h) either stimulated with insulin 100 nM for 15 min or left unstimulated. Data were relativized against insulin (white bar) and expressed as mean ± SEM, N = 4, ***P < 0.001 vs. insulin. B) Western blot of p-IRS-1 (Ser307) and IRS-1 of control, Ruthenium Red (Ru, 10 μM, 30 min prior stimuli) and xestospongin C (XeC, 100 μM, 30 min prior stimuli) treated cardiomyocytes either stimulated with insulin 100 nM for 15 min or left unstimulated. Data were relativized against insulin (white bar) and expressed as mean ± SEM, N = 3, *P < 0.001 vs. Insulin. [file 12964_2014_68_MOESM5_ESM.pdf]
